# Defibrotide for Protecting Against and Managing Endothelial Injury in Hematologic Malignancies and COVID-19

**DOI:** 10.3390/biom15071004

**Published:** 2025-07-14

**Authors:** Edward Richardson, Clifton C. Mo, Eleonora Calabretta, Francesco Corrado, Mehmet H. Kocoglu, Rebecca M. Baron, Jean Marie Connors, Massimo Iacobelli, Lee-Jen Wei, Emily J. Benjamin, Aaron P. Rapoport, Maribel Díaz-Ricart, Antonio José Martínez-Mellado, Carmelo Carlo-Stella, Paul G. Richardson, José M. Moraleda

**Affiliations:** 1 Department of Medicine, Warren Alpert Medical School at Brown University, Providence, RI 02903, USA; 2 Department of Medical Oncology, Dana-Farber Cancer Institute, Jerome Lipper Center for Multiple Myeloma Research, Harvard Medical School, Boston, MA 02215, USA; 3 Department of Biomedical Sciences, Humanitas University, 20089 Milan, Italy; 4 IRCCS Humanitas Research Hospital, 20089 Milan, Italy; 5 Department of Medical Oncology, Dana-Farber Cancer Institute, Boston, MA 02215, USA; 6 Broad Institute of Massachusetts Institute of Technology (MIT) and Harvard, Cambridge, MA 02142, USA; 7 University of Maryland Greenebaum Comprehensive Cancer Center, Department of Medicine, University of Maryland School of Medicine, Baltimore, MD 21201, USA; 8 Transplant and Cellular Therapy Program, University of Maryland Greenebaum Comprehensive Cancer Center, Baltimore, MD 21201, USA; 9 Division of Pulmonary and Critical Care Medicine, Brigham and Women’s Hospital, Harvard Medical School, Boston, MA 02115, USA; 10 Division of Hematology, Brigham and Women’s Hospital, Boston, MA 02115, USA; 11 Techitra S.r.l., 20123 Milan, Italy; 12 Department of Biostatistics, Harvard T.H. Chan School of Public Health, Boston, MA 02115, USA; 13 Hematopathology, Pathology Department, CDB, Hospital Clinic, IDIBAPS, 08036 Barcelona, Spain; 14 Barcelona Endothelium Team, 08036 Barcelona, Spain; 15 Department of Hematology, University Hospital Virgen de la Arrixaca, IMIB-Pascual Parrilla, University of Murcia, 30120 Murcia, Spain; 16 Department of Medicine, Faculty of Medicine, Institute of Biomedical Research (IMIB-Pascual Parrilla), University of Murcia, 30120 Murcia, Spain

**Keywords:** CAR T-cell therapy, bispecific antibodies/bispecific T-cell engagers, COVID-19, CRS, endotheliitis, endotheliopathy, endothelial injury, inflammation, TA-TMA, VOD/SOS

## Abstract

Defibrotide, which is approved for treating hepatic veno-occlusive disease (VOD)/sinusoidal obstruction syndrome (SOS), exhibits pleiotropic anti-inflammatory, anti-thrombotic, and fibrinolytic properties, conferring broad endothelial protective effects. Given these mechanisms, defibrotide has potential utility in various conditions involving endothelial injury or activation. In this review we outline the endothelial-protective mechanisms of defibrotide and comprehensively summarize current evidence supporting its applications in hematologic malignancies, including the prevention and treatment of hepatic VOD/SOS, graft-versus-host disease, and transplant-associated thrombotic microangiopathy. Additionally, we discuss its role in mitigating key toxicities linked to chimeric antigen receptor (CAR) T-cell therapies and bispecific antibodies, such as cytokine release syndrome (CRS) and immune effector cell-associated neurotoxicity syndrome (ICANS). We also explore emerging evidence on defibrotide’s potential in SARS-CoV-2 infection-associated endotheliopathies, including acute COVID-19 and post-acute sequelae of SARS-CoV-2 infection (“long-COVID”), and the endothelial protective activity of defibrotide in these settings. Finally, we highlight potential future applications of defibrotide in hematologic malignancies and viral infections, emphasizing its multimodal mechanism of action.

## 1. Introduction

Defibrotide (DF), a polydisperse oligonucleotide mixture derived from controlled depolymerization of porcine gut mucosa, comprises approximately 90% single-stranded and 10% double-stranded phosphodiester oligonucleotides [1,2]. DF has a broad range of anti-inflammatory, anti-thrombotic, and fibrinolytic properties. Through these mechanisms, DF provides protective effects in multiple settings of endothelial injury or activation. DF is approved in the US and the EU for the treatment of hepatic veno-occlusive disease (VOD)/sinusoidal obstruction syndrome (SOS) [1,2,3]. DF’s range of endothelial protective mechanisms nonetheless extends its utility to other conditions mediated by endothelial dysfunction. These include managing common toxicities associated with immune effector cell therapies—chimeric antigen receptor (CAR) T-cell therapies and bispecific antibodies—used for the treatment of multiple myeloma (MM) and other hematologic malignancies. SARS-CoV-2 infection—acute COVID-19 and post-acute sequelae of SARS-CoV-2 infection (PASC, or “long COVID”)—also features endothelial disruption as a central process of pathobiology, and DF may offer benefits in potentially reversing endotheliitis in these diseases. Here, we review the effects of defibrotide on the endothelium, its protective activity, and potential roles in managing and preventing endothelial damage in patients with hematologic malignancies, COVID-19, and/or PASC.

## 2. Mechanisms of Endothelial Protection with Defibrotide

### 2.1. Functions of the Endothelium and Impacts of Injury/Activation

The endothelium and endothelial cells have a variety of functions that may differ according to tissue location [4,5,6,7]; in the context of this review, the key roles addressed are as follows. The endothelium is critical for the regulation of host defense and inflammation through the expression of adhesion molecules including intercellular cell adhesion molecule 1 (ICAM-1), vascular cell adhesion molecule 1 (VCAM-1), E-selectin, and P-selectin, as well as through cytokine expression, including tumor necrosis factor alpha (TNFα) and interleukin (IL) 1β. Endothelial cells produce and respond to vascular endothelial growth factor (VEGF), basic fibroblast growth factor (bFGF)/FGF2, and angiopoietin-2 (Ang-2) to undergo angiogenesis, the formation of novel vessels from existing ones [8]. The endothelium also contributes to vascular homeostasis through the expression of angiopoietin-1 and 2 (Ang-1/Ang-2) and the receptor tyrosine kinase Tie-2. These mechanisms impact vascular permeability through tight junction modulation, claudin 14, and junctional adhesion molecule (JAM) expression [4,9]. The integrity of the vascular endothelium maintains a selective barrier between the bloodstream and tissues, including the blood–brain barrier protecting the central nervous system (CNS). An additional key role of the endothelium is in hemostasis, as endothelial cells directly regulate coagulation, thrombogenesis, and fibrinolysis; specifically, endothelial cells maintain the intricate balance between the expression/release of procoagulant factors, such as tissue factor (TF), von Willebrand factor (vWF), platelet-activating mediators (e.g., ADP, thromboxane A2), thrombin, and factors VII, VIII, and X, and anticoagulant factors, such as prostacyclin, nitric oxide (NO), protein C receptor, TF pathway inhibitor (TFPI), thrombomodulin (TM), and plasminogen activator inhibitor-1 (PAI-1) [4].

Endothelial activation can occur through various mechanisms, and endothelial dysfunction may occur in the presence of multiple stimuli or noxae [10]. Pathogens, disease states, and drugs (e.g., conditioning regimens, immunotherapeutics) can activate the complement system, which subsequently drives endothelial injury and activates inflammatory and microthrombotic pathways [10]. Repeated exposure to multiple noxae results in dysregulated immune responses and pathologic endothelial activation, owing to dysregulated expression of cellular and soluble signaling mediators [11]. Endothelial dysfunction is effectively a functional imbalance reflected by changes in a number of biomarkers, including increases in markers of inflammation (such as ICAM-1, VCAM-1, E-selectin, TNFα, IL-1, IL-6), changes in markers of vascular tone and homeostatic balance (e.g., increased Ang-2/Ang-1 ratio, VEGFα, fibroblast growth factor 2 (FGF2); reduced NO and prostacyclin), and increases in procoagulant/prothrombogenic markers (including TM, vWF, TF, neutrophil extracellular traps [NETs]). These specific changes differ according to the source of endothelial injury.

Indices for determining endothelial activation, such as the Endothelial Activation and Stress Index (EASIX) and the modified EASIX (mEASIX) [12,13,14,15,16,17,18], use related clinical and laboratory parameters including lactate dehydrogenase (LDH), creatinine, platelet count, and C-reactive protein (CRP). These indices and other markers of endothelial damage have been associated with various different endothelial-related toxicities and sequelae [12], including VOD, transplant-associated thrombotic microangiopathy (TA-TMA), grade 2–4 acute graft-versus-host disease (GvHD) [13,14], cardiac adverse events [15], as well as with non-relapse mortality (NRM) and overall survival (OS) following allogeneic HCT [13,16]. Notably, in an abstract presented at the American Society of Hematology (ASH) 2024 meeting, a Spanish study of 110 patients with lymphoma and MM receiving CAR T-cell therapy showed a positive correlation between both EASIX and mEASIX and biomarkers of endothelial dysfunction, Ang-2 and suppression of tumorigenicity 2 (ST2). Moreover, the study showed significantly higher mEASIX scores in patients with sepsis compared to cytokine release syndrome (CRS) due to CAR T-cell therapy, a distinction that represents a common diagnostic challenge in clinical practice [18].

### 2.2. Mechanisms of Action of Defibrotide

**Figure 1:**
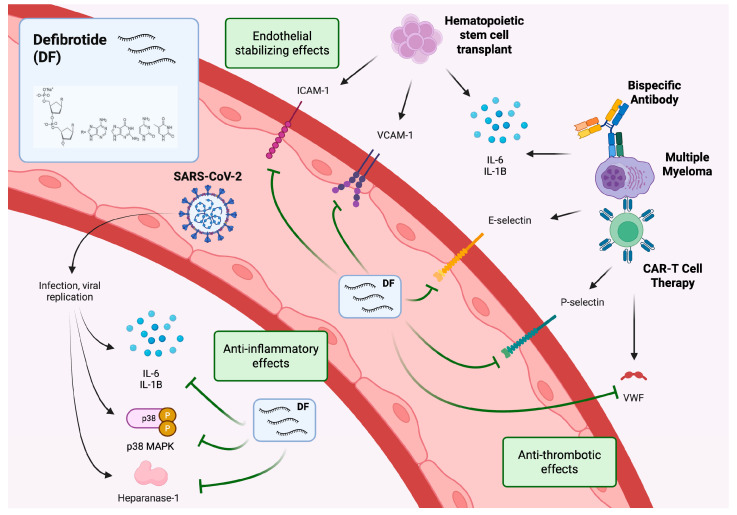
Defibrotide endothelial protective mechanisms in the settings of hematopoietic stem cell transplant, SARS-CoV-2 infection/PASC, and CAR T-cell therapy or bispecific antibody therapy in RRMM. Created in BioRender. Richardson, E. (2025) https://BioRender.com/hh7w5kb. Adapted and updated from Mo CC, et al. Blood Rev;2024;66:101218 [31] and Richardson PG, et al. Bone Marrow Transplant; 2021;56(12):2889–96 [19], under Creative Commons Attribution 4.0 International license (CC BY 4.0; https://creativecommons.org/licenses/by/4.0/, accessed on 19 June 2025). CAR, chimeric antigen receptor; DF, defibrotide; ICAM-1, intercellular adhesion molecule; IL, interleukin; MAPK, mitogen-activated protein kinase; PASC, post-acute sequelae of SARS-CoV-2 infection; RRMM, relapsed/refractory multiple myeloma; SARS-CoV-2, severe acute respiratory syndrome coronavirus 2; VCAM-1, vascular cell adhesion molecule-1; vWF von Willebrand factor.

DF has been shown to modulate multiple markers of endothelial dysfunction and thereby offer treatment of endotheliopathies in a range of scenarios, as reviewed previously (Figure 1) [19,20]. Cellular effects include decreased inflammation through reductions in proinflammatory cytokines such as IL-6, IL-12, TNFα, and thromboxane A2, and increases in anti-inflammatory cytokines including IL-10 and TNFβ. DF reduces cell adhesion by decreasing ICAM-1, VCAM-1, and P/E-selectin expression and increasing prostaglandin I2 and prostaglandin E2; DF also restores thrombo-fibrinolytic balance by reducing vWF, TF, and PAI-1 levels and increasing levels of thrombomodulin and t-PA, as well as enhancing the activity of plasmin [21]. Furthermore, DF reduces endothelial cell activation (reduced PI3K/AKT, vascular E-cadherin [VE-cadherin], p38 MAPK; increased bFGF, VEGF) and maintains vascular tone through the induction of endothelin-1 and increased production of NOS.

DF has been shown to have a protective effect against the proinflammatory and prothrombotic effects of cyclosporine A and tacrolimus plus sirolimus on microvascular endothelial cells, attenuating the increased expression of ICAM-1 and elevated extracellular matrix reactivity [22]. It has also been shown to modulate pathway activation in lipopolysaccharide-activated endothelial cells associated with leukocyte migration and activation, vasculogenesis, and inflammatory responses [23]. Furthermore, through its effects on the PI3K/Akt signaling pathway, DF prevented the upregulation of histone deacetylase expression in human umbilical cord vein endothelial cells following exposure to sera from patients with end-stage renal disease on hemodialysis; DF also inhibited upregulation of endothelial activation markers, including ICAM-1, vWF, and ROS [24].

Related findings from recent studies support and extend these mechanisms of action of DF [25,26,27]. For example, DF has been shown to be effective at suppressing NET formation and venous thrombosis in a mouse model of antiphospholipid syndrome [28,29]. In an abstract presented at the ASH 2024 meeting, it was also shown to increase fibrinolytic activity, as demonstrated by elevated levels of tissue plasminogen activator (tPA) and prostacyclin (PGI_2_), in patients with acute chest syndrome related to sickle cell disease, highlighting its ability to reduce hypercoagulability [30].

## 3. Defibrotide for Managing and Protecting Against Endothelial Injury in Hematologic Malignancies

MM and other hematologic malignancies, such as lymphomas and leukemias, are associated with direct endothelial damage. For example, MM is characterized by elevated markers of inflammation and thrombosis/coagulopathy [32], increased cytokine signaling [31], and dysregulated signaling between MM cells and the bone marrow (BM) microenvironment [33]. Furthermore, several standard-of-care treatment modalities, including hematopoietic cell transplantation (HCT) and certain pharmacotherapies, are linked to the development of endotheliopathies [34,35], for which DF may be utilized as treatment or prophylaxis.

### 3.1. Hepatic VOD/SOS

VOD/SOS is a potentially life-threatening complication of HCT with an overall incidence of 2–14% and up to 60% in high-risk patients [36,37,38,39,40]. The pathophysiology involves primary injury to sinusoidal endothelial cells, hepatocytes, and stellate cells, giving rise to venular microthrombosis, fibrin deposition, ischemia, and fibrogenesis, with major systemic complications including portal hypertension, hepatorenal syndrome, multi-organ dysfunction (MOD), and potentially death [41,42]. The vascular endothelium is the primary target of therapeutic strategies in VOD/SOS, as toxic metabolites from high-dose chemotherapy conditioning directly affect the endothelium, resulting in increased adhesion molecule expression, cytokine signaling, and expression of procoagulant factors such as vWF [43,44,45].

#### 3.1.1. Mechanism of Action of Defibrotide in Hepatic VOD/SOS

DF has demonstrated a range of effects in the setting of hepatic VOD/SOS. It provides endothelial protection through the maintenance of sinusoidal vascular integrity and the reduction in heparanase expression [45,46], and exerts anti-inflammatory effects via reductions in TNFα, VCAM-1, and p38 mitogen-activated protein kinase (MAPK) as well as Akt phosphorylation [45,47,48,49]. Additionally, DF helps restore the thrombotic–fibrinolytic balance through reductions in TF and PAI-1 and augmented t-PA activity [45,50,51,52]. DF (but not low-molecular-weight heparin [LMWH]) has also been shown to prolong survival in a rat model of monocrotaline (pyrrolizidine alkaloid)-induced SOS and to reduce levels of TNFα and PAI-1 [53].

#### 3.1.2. Defibrotide for Managing Hepatic VOD/SOS

Multiple clinical studies and real-world analyses have demonstrated the efficacy of DF for the management of hepatic VOD/SOS (Table 1) [39,54,55,56,57,58,59,60,61,62,63,64,65,66,67,68,69,70,71,72,73,74,75,76,77,78,79,80,81]. The severity of VOD/SOS is variable; however, historical mortality in severe cases with MOD prior to DF treatment exceeded 80% [36]. As outlined below, numerous studies of DF in VOD/SOS present a marked reduction in this figure. Based on its demonstrated efficacy, DF is a cost-effective option for treating severe VOD/SOS with MOD [82].

A previous systematic review included findings from 17 prospective and retrospective studies for a total of 2598 patients with hepatic VOD/SOS treated with DF [83]. The analysis yielded a pooled day +100 survival rate of 54% overall and 56% in patients receiving standard DF dose of approximately 25 mg/kg/day. Overall, 1260 of the 2598 patients had MOD. The day +100 survival rates were 44% and 71% in patients who received the standard DF dose, with or without MOD, respectively. Respective rates in pediatric and adult patients treated with the standard DF dose were 68% and 48% [83]. A second systematic review and meta-analysis involving 3002 patients treated with DF also showed the efficacy of DF in this setting, with a complete resolution (CR) rate and a day +100 survival rate in the whole cohort of 57% and 58% and in the group with severe VOD of 39% and 44%, respectively [84].

These findings were mirrored in a separate pooled analysis of three studies involving 651 patients with MOD post-HCT, including 233 (36%) with ventilator and/or dialysis dependence [85]. The data showed that, while the overall day +100 survival rate was 44% in this population (*n* = 651), survival rates were higher in those with less severe MOD: 48% in patients without ventilator or dialysis dependence (*n* = 418) versus 40% in those with one dependence (*n* = 137), 33% in patients with one or both (i.e., ventilator and/or dialysis) dependencies (*n* = 233), and 28% in those with both ventilator and dialysis dependence (*n* = 96). Nevertheless, these day +100 survival rates were higher than those in a historical control population with VOD/SOS and MOD, in which the overall rate was 25% and the rates were 28% and 14%, respectively, in patients with no dependence versus one dependence [85].

A further pooled analysis from the same three studies (incorporating 1176 patients), demonstrated the importance of continuing DF until VOD/SOS resolution [86]. Among all 390 patients who achieved CR and had data available, the median time to DF discontinuation due to CR was 22–24.5 days, and discontinuation beyond 28 days occurred in 15–40% of patients, highlighting the benefit of continuing DF treatment past the recommended minimum of 21 days. Importantly, day +100 survival was significantly higher in those who discontinued DF due to a CR compared to those who did not (92.5% vs. 37.3%), further emphasizing the value of treatment continuation to achieve CR [86].

As well as these meta-analyses and pooled analyses, the efficacy and effectiveness of DF have been shown in multiple key individual clinical studies and real-world evaluations. For example, in the pivotal phase 3 trial of DF versus historical controls in patients with post-HCT severe VOD/SOS with renal and/or pulmonary failure (MOF) [54], 102 patients were enrolled to the DF arm and compared with 32 matched contemporaneous and validated historical controls; 43% and 44% were pediatric patients, and 88% and 84% had received an allogeneic HCT. The study illustrated the challenges of undertaking a comparative trial in a rare and complex disease state, with the contemporaneous control group requiring review of 6867 patient medical charts to obtain 32 patients with an unequivocal diagnosis of VOD/SOS with MOF; with the originally planned sample size of 80 patients in the control group proving to be not feasible, statistical analysis adjustment was required. Compared to historical controls, patients receiving DF showed a significant improvement in day +100 survival rate (38.2% vs. 25.0%), the primary endpoint of the study. The estimated between-group difference (stratified by propensity score quintile via the Koch method) was 23% (95% CI 5.2–40.8, *p* = 0.0109). The observed rate of CR at day +100 was also significantly greater, at 25.5% vs. 12.5%. Notably, toxicity was generally manageable with DF, with lower rates of diarrhea, hemorrhagic adverse events (AEs), hypotensive AEs, and coagulopathy than in historical controls, and with only 11% of patients discontinuing due to possible DF-related AEs [54]; indeed, while the DF prescribing information includes a warning to monitor patients for bleeding and a contraindication for concomitant administration with systemic anticoagulant or fibrinolytic therapy [1], DF does not appear to significantly increase overall rates of hemorrhage in VOD/SOS [19,87], and a meta-analysis of DF studies in the non-VOD/SOS setting has demonstrated a reduced risk of bleeding events compared with controls (risk ratio 0.36) [88]. Results from an expanded-access study of DF in 1000 patients who had post-HCT VOD/SOS, including 512 with MOD, were supportive of the phase 3 trial findings [55]; overall day +100 survival rate was 58.9%, including 49.5% in patients with MOD and 68.9% in those without, and 47.1% and 67.9% in adult and pediatric patients, respectively.

Among the real-world studies demonstrating the effectiveness of DF for VOD/SOS [56,57,68], the Dana-Farber Cancer Institute (DFCI)/Brigham and Women’s Hospital (BWH) experience in 28 patients with VOD/SOS post-allogeneic HCT showed complete resolution of VOD/SOS in 75% and a day +100 survival rate of 64%, including 57% in patients with MOD [57]. Similarly, a DEFIFrance registry study in 251 patients with severe/very severe VOD/SOS demonstrated a CR rate of 55%, including 84% and 46% in pediatric and adult patients, respectively, a day +100 CR rate of 74%, 84% in severe and 63% in very severe VOD/SOS cases, and a day +100 survival rate of 61% [56].

An important element of DF treatment for VOD/SOS is prompt diagnosis and initiation of therapy [38,89,90], which has been shown to offer improved outcomes compared with delayed DF treatment [91,92]. In an analysis of data from 573 patients on the DF expanded access protocol, 31.9% received DF on the day of VOD/SOS diagnosis and 93.0% had started DF by day +7 [92]. Day +100 survival rate differences were 8.8% between patients starting DF on day 0/1 versus >1, 22.1% between patients starting DF on day ≤2 versus >2, and 20.3%, 20.2%, and 20.9% for subsequent cut-offs of day ≤3, ≤4, and ≤7, respectively. Similar findings were seen in patients with MOD. Supportive findings were seen in a multivariate analysis of a retrospective multicenter study, in which early DF intervention was the only factor associated with a CR [62] and in a single-center analysis of 111 pediatric patients who underwent HCT, in which early versus non-early DF intervention significantly reduced the peak grade of VOD/SOS [91]. Further, in a Korean retrospective real-world analysis, DF intervention within 2 days of VOD/SOS diagnosis resulted in a higher CR rate (55.6% vs. 30.4%) and better day +100 survival (37.0% vs. 26.1%) [79].

#### 3.1.3. Defibrotide Prophylaxis Against Hepatic VOD/SOS

DF has also been evaluated as prophylaxis in HCT recipients for preventing hepatic VOD/SOS (Table 2) [66,80,81,93,94,95,96,97,98,99,100,101,102,103,104,105,106,107]. Of particular note, in a phase 3 trial of DF versus controls in 356 pediatric patients who had received myeloablative conditioning (MAC) for autologous or allogeneic HCT [93], the primary analysis demonstrated a rate of VOD/SOS at day +30 of 12.2% vs. 19.9%, a reduction of 7.7 percentage points (95% CI for risk difference, –15.3% to –0.1%, *p* = 0.049). This risk difference was –5.9% to –13.0% when evaluated separately in infants, children, and adolescents. Importantly, the safety profile showed no increase in cumulative hemorrhagic incidence and numerically lower rates of TA-TMA (3% vs. 4%) and fatal infections/infestations (3% vs. 6%) in patients receiving DF prophylaxis. Supportive findings were provided by a meta-analysis of 20 studies of DF as VOD/SOS prophylaxis [94]; in an analysis of 3005 patients, incidence of VOD/SOS with DF prophylaxis was 5% (5% in adults, 8% in pediatric patients), whereas incidence in controls (from comparative studies) was 16%, showing a risk ratio for developing VOD/SOS of 0.30 (95% CI 0.12–0.71, *p* = 0.006). Similarly, a network meta-analysis of primary prophylaxis options for VOD/SOS in patients receiving cell-based therapies showed an odds ratio of 0.64 for the development of VOD/SOS in those receiving DF prophylaxis, with a greater magnitude of benefit (odds ratio 0.51) in a subgroup analysis of patients receiving allogeneic HCT [107].

In contrast, the phase 3 HARMONY trial of DF versus best supportive care (BSC) in 372 adult and pediatric patients receiving SCT showed no difference in day +30 VOD/SOS-free survival (67% vs. 73%), and rates of VOD/SOS occurrence at any time of 14% versus 18% [95]. Interestingly, in an abstract presented at the ASH 2024 meeting, a retrospective analysis of prophylactic DF that stratified high-risk pediatric patients according to the HARMONY trial criteria similarly found no benefit in VOD/SOS prevention [108]. However, several potential issues were identified with regard to the HARMONY trial that could have contributed to the failure to demonstrate efficacy of DF as prophylaxis [109]. First, HARMONY included a broad patient population, with a limited representation of very high-risk pediatric patients. Second, with regard to the study design, the power calculation and sample size were insufficient based on an overestimate of VOD/SOS incidence in the enrolled population; for the primary endpoint of VOD/SOS-free survival at Day 30, only 7% of the trial population were at risk for this composite endpoint. Furthermore, the study design allowed for DF use for emergent VOD/SOS in the BSC group. Finally, there were discrepancies in VOD/SOS diagnosis between investigators and central adjudication employed in the trial. HARMONY thus provides further illustration of the challenges of designing and conducting comparative trials with appropriate statistical powering in a complex disease state such as VOD/SOS that is a relatively uncommon outcome. Nevertheless, despite findings from the HARMONY trial, collective evidence suggests that DF may have a beneficial impact when used as prophylaxis against VOD/SOS.

### 3.2. Graft-Versus-Host Disease

Acute GvHD is a complication of allogeneic HCT mediated by alloreactive T cells in the donor graft, which recognize mismatched HLA antigens on endothelial cells, leading to endothelial damage, a key component of the pathophysiology of the condition [110]. High-dose chemotherapy conditioning induces systemic inflammation and endothelial cell damage, and endothelial cells are activated by inflammatory cytokines and damage-associated molecular patterns (DAMPs) through toll-like receptor (TLR) signaling. This activation promotes the expression of adhesion molecules, facilitating the recruitment of innate and adaptive immune cells to sites of inflammation. In the lymph nodes, host dendritic cells (DCs) present allogeneic peptides, leading to the activation of CD8+ and CD4+ T cells. Cytotoxic CD8+ T cells directly damage endothelial cells, while CD4+ T cells release inflammatory cytokines, such as TNFα and interferon-γ, further activating endothelial cells. TLR signaling via MAPKs upregulates the expression of adhesion molecules (selectins, integrins), enhancing leukocyte transmigration. Simultaneously, TNFα receptor signaling on endothelial cells increases Ang-2 expression and permeability. These mechanisms collectively contribute to the progression of GvHD in target organs.

#### Defibrotide for Treating or Preventing GvHD

Evidence from preclinical studies supports the potential of DF for the prevention of GvHD as well as the mechanistic rationale underlying its effects [111]. Notably, in mice receiving fully MHC-mismatched allogeneic HCT, prophylactic or therapeutic administration of DF was effective in preventing T cell and neutrophil infiltration as well as acute GvHD-related tissue-specific damage in the skin, liver, colon, and tongue. Additionally, DF treatment restored the balance of inflammatory cytokines. These effects resulted in a reduction in the incidence and severity of acute GvHD, significantly improving animal survival [111]. Furthermore, mice with acute GvHD exhibited elevated levels of proinflammatory cytokines—including interferon-γ, TNFα, IL-6, and IL-12—alongside decreased concentrations of anti-inflammatory cytokines such as TGFβ and IL-10 on day +10 post-HCT. In contrast, mice receiving prophylactic DF showed significant reductions in pro-inflammatory mediators and increased levels of anti-inflammatory cytokines compared to untreated controls [111]. Similarly, in a recently published study using a murine model of allogeneic HCT, DF treatment improved survival and reduced clinical GvHD by exerting anti-inflammatory and endothelial protective effects, as evidenced by lower levels of TNFα, IL-6, VCAM-1, ICAM-1, and Ang-2 [112]. Moreover, in vitro studies using endothelial cell lines exposed to sera from patients with acute GvHD showed that DF suppressed markers of vascular angiogenesis and endothelial activation driven by GvHD-associated patient sera [113].

Clinical studies have also demonstrated the efficacy of DF for the prevention of GvHD. In a prespecified secondary/exploratory analysis of DF for the prevention of GvHD, as part of the phase 3 pediatric VOD/SOS prophylaxis study, DF significantly reduced the rate of acute GvHD by day +30 (34% vs. 52%) and by day +100 (47% vs. 65%), with significantly lower incidence and severity (grades 1–4) compared with controls (incidence *p* = 0.0057 and severity *p* = 0.0062 at 30 days; incidence *p* = 0.0046 and severity *p* = 0.0034 at 100 days), even when grade 1 acute disease was excluded [93]. Similarly, in a phase 2 study of DF plus standard-of-care treatment versus standard-of-care treatment alone for GvHD prophylaxis in 152 patients receiving allogeneic HCT, the cumulative incidence of grade B–D acute GvHD by day +100 post-transplant was 38.4% vs. 47.1% (37.0% vs. 45.7% in a sensitivity analysis using disease relapse as a competing risk) [114].

A retrospective analysis in 38 adult patients receiving allogeneic HCT showed that DF in combination with other immunosuppressive agents (rabbit anti-T lymphocyte globulin, post-transplant cyclophosphamide, cyclosporine) may decrease the risk of GvHD—the 1-year cumulative incidence of grade III-IV acute GvHD and moderate/severe chronic GvHD were 20.6% and 5.3%, respectively [115]. These findings suggest DF might complement other prophylactic strategies, such as post-transplant abatacept. Further, a retrospective analysis of 47 vs. 44 pediatric allogeneic HCT recipients who did versus did not receive DF prophylaxis showed a significantly lower rate of acute GvHD (23% vs. 50%, including 4% vs. 39% grade II–IV); the odds ratio for developing acute GvHD with DF prophylaxis was 0.31 overall and 0.11 for moderate/severe GvHD [116]. Notably, levels of proinflammatory cytokines were significantly lower in the DF prophylaxis versus control group. Similarly, rates of acute GvHD were lowered with the use of DF in a Turkish analysis of 195 consecutive adult patients receiving allogeneic HCT [117]; in patients receiving DF prior to HCT (concurrently with conditioning), DF post HCT, or no DF, the overall rate of acute GvHD was 25.5%, 40%, and 46.5%, respectively, and the rate of grade III-IV acute GvHD was 0%, 11.2%, and 15.5%. Conversely, however, there have been other studies that have not demonstrated a benefit from DF prophylaxis on the occurrence of acute GvHD [118].

### 3.3. Transplant-Associated Thrombotic Microangiopathy

TA-TMA is associated with abnormal endothelial cell activation, complement activation, platelet-rich thrombi formation, and microvascular hemolytic anemia, ultimately leading to end-organ dysfunction [119]. It occurs following both autologous and allogeneic stem cell transplantation but is frequently observed after allogeneic transplantation. Endothelial injury caused by high-dose conditioning regimens and calcineurin/mammalian target of rapamycin (mTOR) inhibitors for GvHD prophylaxis results in elevated levels of proinflammatory cytokines (e.g., IL-2, TNFα), procoagulant factors (e.g., vWF, TF, factor VIIa), and soluble adhesion molecules, which perpetuates the activation of the complement cascade. Development of NETs following endothelial cell damage may represent a specific mechanism of complement activation in TA-TMA. Additionally, nitric oxide (NO) depletion impairs vasodilation, promoting platelet aggregation and the development of microthrombi. Of note, in the context of MM treatment, TMA has also been associated with the use of the proteasome inhibitor (PI) carfilzomib, although the precise mechanisms underlying PI-induced TMA have not been fully elucidated [120].

#### Defibrotide for Treating or Preventing TA-TMA

Data from a few small studies or retrospective analyses suggest that DF has activity as a treatment for or prophylaxis against TA-TMA [121,122]. In a European Society of Blood and Marrow Transplantation retrospective study of 17 adults and 22 pediatric patients with TA-TMA who received DF, TA-TMA resolved in 77% of cases, with earlier diagnosis and treatment with DF associated with higher resolution rates [123]. An Indian retrospective case series of three patients who had TA-TMA after allogeneic HCT for AML, CML-AP, or Pro-B ALL demonstrated that low-dose DF administered for 7–19 days resulted in resolution or improvement of TA-TMA in all cases [124]. A pilot study of DF prophylaxis in 25 high-risk pediatric patients, 14 of whom were receiving tandem autologous HCT for neuroblastoma and 11 of whom were undergoing allogeneic HCT, identified only one case (4%) of non-severe TA-TMA, occurring 12 days post-HCT. This incidence was significantly lower than the historical rate of TA-TMA of 18–40% in autologous/allogeneic HCT patients [125]. Additionally, a retrospective analysis of 31 patients with TA-TMA who were treated with DF ± plasmapheresis ± rituximab showed a 61% overall response rate (100% in low-risk, 25% in high-risk patients), although outcomes were poor [126].

### 3.4. Defibrotide for Treating or Preventing Idiopathic Pneumonia Syndrome (IPS)

IPS is a non-infectious acute lung injury condition occurring post-HCT [127]. Pulmonary dysfunction in IPS and acute respiratory distress syndrome (ARDS) is mediated, at least in part, by pulmonary endothelial cell injury and activation. In mouse models of IPS and lipopolysaccharide-induced ARDS, DF has been shown to substantially modulate endothelial cell injury, with reduced expression of TNFα, Ang-2, E-/P-selectin, and IL-6 [127]. Further clinical studies are warranted to investigate a potential clinical role in IPS treatment or prophylaxis.

### 3.5. Immune Effector Cell Therapy-Associated Cytokine Release Syndrome and Neurotoxicity

CAR T-cell therapies are among the standard-of-care (SOC) therapies for relapsed/refractory multiple myeloma (RRMM), as well as for leukemias and lymphomas [128]. For RRMM, the BCMA-directed therapy idecabtagene vicleucel (ide-cel) is approved for use after ≥2 prior lines, including a PI, an immunomodulatory drug (IMiD), and a CD38 monoclonal antibody (mAb) [129], while ciltacabtagene autoleucel (cilta-cel, also targeting BCMA) is approved for patients who have received ≥1 prior line, including a PI and an IMiD, and who are refractory to lenalidomide [130]. There are also several US FDA-approved CD19-directed CAR T-cell therapies for leukemia and lymphoma [131]. For large B-cell lymphoma (LBCL)/diffuse LBCL (DLBCL), axicabtagene ciloleucel (axi-cel) is approved for patients who are refractory or relapsed within 12 months of first-line chemoimmunotherapy [132], and tisagenlecleucel (tisa-cel) is approved for patients who have received ≥2 prior lines [133]. Axi-cel and tisa-cel are also approved for the treatment of patients with relapsed or refractory follicular lymphoma (FL) after ≥2 lines of therapy [134,135]. Brexucabtagene autoleucel (brexu-cel) is approved for the treatment of relapsed or refractory mantle cell lymphoma (MCL) [136], while lisocabtagene maraleucel is approved for the treatment of relapsed or refractory FL, LBCL, and MCL [137]. Additionally, tisa-cel and brexu-cel have received approval for the treatment of relapsed/refractory B-cell precursor acute lymphoblastic leukemia (ALL) in patients aged ≤25 years and adults, respectively [138,139]. Obecabtagene autoleucel is also approved for relapsed/refractory adult B-cell precursor ALL [140], and lisocabtagene maraleucel is approved for relapsed/refractory chronic lymphocytic leukemia [137]. An important element of treatment with autologous CAR T-cell therapies is that they typically require a manufacturing time of approximately 4–10 weeks. During this period patients receive bridging therapy to control or reduce their disease, followed by lymphodepleting therapy with, for example, fludarabine [141]. This may result in additional endothelial injury and predispose patients to subsequent adverse effects.

A number of bispecific antibodies/T-cell engagers are also approved for the treatment of later-relapse RRMM, including the BCMA-targeted agents teclistamab [142] and elranatamab [143] and the GPRC5D-targeted agent talquetamab [144], with several others with similar, different, or multiple targets under investigation. These dual-specific antibodies facilitate cell-to-cell interactions between MM cells expressing tumor-specific antigens and patients’ T cells via engagement of CD3 (xCD3), leading to selective cell lysis [145]. Additionally, several bispecific antibodies are approved for the treatment of non-Hodgkin’s lymphoma and leukemia [146,147]; these include the CD20-targeted agents epcoritamab for DLBCL and FL, glofitamab for DLBCL and LBCL arising from FL, and mosunetuzumab for FL, as well as the CD19xCD3 agent blinatumomab for B-cell precursor ALL.

Among the common AEs associated with these immune effector cell therapies are the endotheliopathy-related toxicities of CRS and immune effector cell-associated neurotoxicity syndrome (ICANS) [31,148]. The pathogenesis of CRS involves the interaction of CAR T cells with MM cells, or the engagement of MM cells and T cells by the bispecific antibody, which results in T-cell cytokine production, as well as macrophage activation and further production of proinflammatory cytokines [149]. The cytokine storm results in endothelial activation, with major inflammatory effects mediated by TNFα, IL-6, IL-1β, interferon-γ, and potentially through NET formation, as well as increases in coagulation markers such as Ang-2 and vWF. With CAR T-cell therapy, more severe CRS has been associated with fludarabine exposure prior to CAR T-cell infusion, which potentially augments endothelial injury. The pathophysiology of ICANS is similarly driven by CAR T-cell/T-cell interaction with MM cells, with endothelial activation following CAR T-cell activation and cytokine release likely increasing blood–brain barrier permeability [149]. This can lead to elevated cytokine levels in the cerebrospinal fluid and CNS, thereby driving neuroinflammation and associated neurotoxicity. Notably, a range of studies have shown that these toxicities are associated with markers of endothelial activation at baseline or during treatment [31]. The recommended management of CRS and ICANS includes anti-cytokine agents such as tocilizumab (IL-6 receptor antagonist), anakinra (IL-1 receptor antagonist), siltuximab (anti-IL-6 mAb), etanercept (TNFα inhibitor), and infliximab (anti-TNFα mAb) [150,151]. Hemophagocytic lymphohistiocytosis (HLH) is another CAR T-cell therapy-related severe adverse effect and is associated with endothelial dysfunction leading to uncontrolled endotheliitis, likely a result of a high-cytokine milieu (primarily interferon-γ). Treatment options for HLH overlap with those for CRS and ICANS and additionally may include agents such as emapalumab (anti-interferon-γ mAb) [152].

#### Defibrotide for Treating or Preventing Immune Effector Cell Therapy-Associated CRS and Neurotoxicity

A phase 2 study has evaluated DF for preventing CAR T-cell therapy-associated neurotoxicity in 25 patients receiving axi-cel for relapsed/refractory diffuse large B-cell lymphoma (DLBCL) [153]. Patients received DF for 3 days in tandem with lymphodepletion therapy and then from day 0 to day +7 after axi-cel. For the primary endpoint of the day +30 rate of neurotoxicity, DF demonstrated a numerically (but not statistically significantly) lower incidence of any-grade neurotoxicity of 50% (25% grade ≥ 3), compared with the reference rate of 64% seen in the ZUMA-1 trial of axi-cel in B-cell lymphoma. DF as a potential preventive strategy for CRS/ICANS warrants further exploration (Figure 1), including the potential benefit of combining DF with IL-6 or IL-1 blockade in more severe cases.

## 4. Defibrotide for Managing and Protecting Against Endotheliopathies Associated with COVID-19

### 4.1. Endotheliopathies Associated with SARS-CoV-2 Infection Resulting in COVID-19 and PASC

Endothelial dysfunction is a hallmark of the pathobiology of SARS-CoV-2 infection, driving COVID-19 morbidity and mortality via cytokine release, coagulopathy, and microvascular injury [154,155,156,157]. Direct infection of endothelial cells with SARS-CoV-2 occurs through ACE-2 endothelial receptors, and infection can cause endotheliitis resulting in apoptosis, with endothelial dysfunction propagated by cytokine release following infection, leading to the activation of coagulation and inflammation. Subsequent effects include increased P-selectin and vWF expression leading to platelet activation, accumulation, and production, followed by VEGF and TF release, and complement activation and increased expression of leukocyte adhesion molecules (ICAM-1, VCAM-1, E-selectin) promoting inflammation and amplifying pathologic cytokine production. Excess cytokines subsequently impair endothelial barrier functions, as IL-1β and TNFα expression promotes the loosening of inter-endothelial junctions and associated vascular leakage. Increased IL-6, IL-8, and TNFα also drive the production and release of vasoactive molecules such as thrombin, thromboxane A2, and VEGF. Following the acute phase, viral dissemination into tissue reservoirs can result in persistent residual inflammation and prolonged endothelial activation, as seen with PASC [31,158].

For patients with hematologic malignancies, there are multiple potential sources of endothelial injury in the context of endemic COVID-19 and ongoing waves of SARS-CoV-2 infections [31], including: from the malignancy itself, as in MM-associated endotheliopathy; from injury associated with commonly used treatments, such as autologous HCT in transplant-eligible newly diagnosed MM patients, CAR T-cell therapies in the early-relapse setting, and bispecific antibodies in the later-relapse setting; and from new or previous SARS-CoV-2 infections, for which patients with hematologic malignancies are at elevated risk. Indeed, one retrospective study has suggested that patients who underwent HCT following a SARS-CoV-2 infection had a significantly higher rate of TA-TMA, and a trend for higher rates of VOD/SOS and engraftment syndrome, compared with historical controls, indicating an elevated risk for endothelial-related complications post-infection [159].

### 4.2. Defibrotide for Endothelial Protection in the Setting of COVID-19

There are a number of proposed mechanisms for the potential endothelial protective activity of DF in acute COVID-19 [20,157,160,161,162] and in PASC [158] (Figure 1). DF may counteract the endothelial effects of SARS-CoV-2 infection through increased t-PA and TM expression, decreased vWF and PAI-1 expression, and platelet adhesion inhibition via increases in NO, prostaglandin I2 (PGI2), and prostaglandin E2 (PGE2). DF may also offer anti-inflammatory properties via inhibition of the p38 MAPK pathway, attenuating release of inflammatory mediators including IL-6, thromboxane A2, leukotriene B4, TNF-alpha, and ROS. DF may also inhibit expression and activity of heparanase, modulate endothelial cell injury by downregulating expression of endothelial cell adhesion molecules such as E-selectin, VCAM-1, and ICAM-1, and increase endothelial cell release of anti-inflammatory cytokines.

Preclinical findings support these proposed mechanisms and the role of DF in reversing the endotheliitis of COVID-19. As noted previously, DF results in significant decreases in proinflammatory cytokines in mice undergoing allogeneic HCT. DF has also been shown to significantly reduce the levels of adhesion molecules, including E/P-selectin, VCAM-1, and ICAM-1 [111]. Additionally, in an analysis of human dermal microvascular endothelial cells exposed to plasma from patients with acute COVID-19, DF suppressed cellular pathways associated with endothelial activation by COVID-19 plasma, including TNFα signaling, IL-17 signaling, endothelin activity, and fibrosis [9]. Further exploration of DF and other commonly used anti-inflammatory modalities for COVID-19, such as steroids and IL-6 inhibition, is warranted to determine whether their mechanisms of action may be complementary.

### 4.3. Clinical Findings Demonstrating the Role of Defibrotide for Endothelial Protection in the Setting of COVID-19

A safety study at the University of Michigan investigating the role of DF in the management of SARS-CoV-2-related acute respiratory distress syndrome (ARDS) suggested beneficial effects from a 7-day course of DF [163]. Among 12 patients, 10 of whom required mechanical ventilation and 6 vasopressor support at study entry, a pulmonary response at day 7 was seen in 4 patients, with D-dimer levels decreased within the first 72 h of receiving DF. For example, one patient requiring mechanical ventilation at study entry was extubated on study day 4 following DF treatment, with subsequent removal of all supplemental oxygen on day 7. Day 30 all-cause mortality was 17%, and nine patients remained alive at 64–174 days after starting DF, indicating a 75% survivorship; for context, historical 28-day mortality rates at the time for patients with SARS-CoV-2 ARDS were 26–61.5% [163]. No hemorrhagic or thrombotic complications occurred during therapy.

A phase 2 study in Italy evaluated DF in 48 patients with COVID-19 pneumonia receiving non-invasive ventilation and compared the outcomes with 153 matched case-controls [164]. All 48 patients had a WHO score of 5 on day 1. No significant hemorrhagic or bleeding episodes occurred during the study therapy. There was a trend towards longer OS and respiratory failure-free survival in the DF vs. case-control cohort on adjusted analysis and in a survival prediction model versus SOC management. DF also resulted in a significantly greater mean number of post-recovery days; i.e., in the number of COVID-19-free days out of a predefined 28-day window: 11.60 days with DF, compared with 5.29 days in the case-control observational cohort and 7.99 days predicted for SOC management.

Similarly, in an abstract presented at the 15th Congress of the European Association for Clinical Pharmacology and Therapeutics in 2022, a Spanish phase 1/2 study of DF in 150 patients with WHO grade 4–5 (72%) or 6 (28%) COVID-19 reported data consistent with the known favorable safety profile of DF in VOD/SOS and a preliminary mortality rate due to severe COVID-19 of 27%, which compares with expected mortality of >50% in historical controls [165]. An ongoing DFCI/BWH study in 39 patients (including 6 with MM) to date also confirmed the safety of DF in this setting, and preliminary analysis indicated a favorable impact of DF on cytokine markers and markers of endothelial stress (NCT04652115; Richardson PG, personal communication). In a report on two patients with pediatric inflammatory multisystem syndrome temporally associated with severe SARS-CoV-2 infection (PIMS-TA), DF was shown to be an effective treatment for the syndrome, reducing inflammation and restoring the thrombo-fibrinolytic balance [166]. Additionally, a pilot study of home-administered thromboprophylaxis in patients with COVID-19 and mild-to-moderate symptoms demonstrated that DF and LMWH were equally effective at preventing DVT and thrombotic disease and delivered similarly improved outcomes compared to a control group receiving standard management [167].

Furthermore, a recent report demonstrated the benefit of DF in two patients with RRMM and severe COVID-19 after CAR T-cell therapy [168]. Both patients had severe COVID-19 shortly after receiving CAR T-cell therapy, and experienced prolonged stays in the intensive care unit, with progressive, worsening disease despite maximal standard of care. Both patients experienced rapid improvements in their clinical condition after starting DF for 7–14 days, with intubation avoided; DF resulted in the suppression of SARS-CoV-2-induced non-specific inflammatory response and related CRS, no negative impact on adaptive virus-specific antibody and/or T-cell responses, and no negative impact on persistence of CAR T cells. In both patients, their MM remains in deep and sustained remission.

## 5. Conclusions and Next Steps for Defibrotide

DF’s pleiotropic mechanisms of action—spanning anti-inflammatory, antithrombotic, and fibrinolytic effects—support its role as a versatile endothelial protectant across multiple pathologies. DF is active in modulating and reversing the inflammatory and thrombotic/coagulation pathways that are activated following endothelial injury. DF has also shown endothelial protective effects via these mechanisms of action, through prevention of the cytokine storm that can arise following endothelial exposure to various noxae and maintenance of the thrombotic–fibrinolytic balance.

DF is approved for the management of VOD/SOS in the US and Europe and has also shown efficacy in numerous other conditions associated with endothelial injury following autologous or allogeneic HCT. Despite findings from the phase 3 HARMONY trial, DF may offer benefit as prophylaxis for VOD/SOS, particularly in high-risk cases. DF has demonstrated activity in preventing GvHD following allogeneic HCT and potentially in treating or preventing TA-TMA. Furthermore, DF has shown potential in protecting against the progressive endothelial damage that occurs in sepsis-associated organ dysfunction [169] and for reducing hypercoagulability due to loss of endothelial integrity in patients with sickle cell disease-related acute chest syndrome [30]; recently, DF has generated interest, based on its mechanisms of action, as a novel therapeutic for the key toxicities—CRS and ICANS—associated with CAR T-cell therapy and bispecific antibody therapy, and also for treating endothelial dysfunction associated with SARS-CoV-2 infection.

Based on the effects demonstrated in several small studies/case series, further evaluation of DF is warranted in multiple clinical settings. DF should be further investigated as prophylaxis against or as treatment for CRS and ICANS in high-risk patients with MM, lymphoma [148], and leukemia [12] receiving CAR T-cell or bispecific antibody therapy. Attention should be paid to the timing of DF initiation, as greater efficacy has been observed with earlier vs. later DF initiation in the treatment of VOD/SOS; additionally, combination therapies, such as with anti-IL-6 mAbs, may be beneficial in more severe cases. DF is also being studied as a treatment option for severe COVID-19, in patients with ARDS (NCT04652115), and as prophylaxis against, and treatment of, PASC, and warrants investigation in other conditions characterized by endothelial injury or dysfunction as a central component of pathobiology, as originally proposed in peripheral blood and marrow stem cell transplantation [170]. These settings might include the following:

- Other viral or infectious causes of severe acute lung injury, e.g., serious influenza;
- Inflammatory lung conditions such as IPS or other non-HCT-related lung injury;
- Prevention of microvascular ischemia and thrombosis in ischemic diseases (cardiovascular, neurological);
- Immune-mediated endothelial injury, including autoimmune diseases and antiphospholipid syndrome;
- Solid organ transplant-associated endothelial dysfunction, including ischemia-reperfusion injury and chronic allograft vasculopathy.

Treatment options that offer endothelial protection in these settings will become increasingly important for patients at risk of endothelial injury from multiple sources. This is particularly relevant given the expanding use of CAR T-cell therapy and bispecific antibodies in MM and other hematologic malignancies, the ongoing potential effects of endemic COVID-19 and possible serial waves of SARS-CoV-2 infections as well as other viruses, the growing number of patients with PASC, and the potential threats from emerging viral illnesses.

## Figures and Tables

**Table 1:** Clinical trials and real-world studies of DF treatment for hepatic VOD/SOS.

Study	Patients	VOD/SOS Severity	CR Rate	Survival	Safety
Clinical Trials					
Phase 3 [54]	102 (DF) vs. 32 matched controls	Severe VOD/SOS with renal and/or pulmonary failure	Day +100: 25.5% vs. 12.5%	Day +100: 38.2% vs. 25.0%	Any hemorrhagic AEs 64% vs. 75% Any hypotensive AEs 39% vs. 50% Diarrhea 23.5% vs. 37.5% Coagulopathy 2% vs. 15.6% Possible DF-related AE leading to discontinuation 11%
Randomized dose-finding trial [70]	75 lower-dose DF/ 73 higher-dose DF	Severe VOD/SOS	Day +100: 46% (49%/43%)	Day +100: 42% (44%/39%)	TRAEs 8% Grade 3/4 expected AEs 54% Grade 3–5 renal failure 31%, hypotension 29%, hypoxia 26%, other pulmonary 22%
EBMT prospective observational study [69]	104	62 with severe VOD/SOS	Day +100: 73% MOD/MOF resolution in 53%	Day +100: 73%	SAEs 32% Infection 24% Bleeding 13%
Real-world studies					
Expanded access study [55]	1000 with post-HCT VOD/SOS	512 with MOD	NR	Day +100: 58.9% 49.5% with MOD 68.9% without MOD 47.1% adults 67.9% pediatric	SAEs 53.7% TRAEs 21.0% Hemorrhage 29.0% Hypotension 12.0%
International CUP [61]	710	41% with MOF 48% severe VOD/SOS	NR	Day +100: 54% 40% with MOF 65% without MOF 46% adult 65% pediatric	AEs 53% SAEs 51% Withdrawal due to AE 9% Sepsis 7% GI hemorrhage 3%
Spanish GETH/GETMON analysis [66]	253 pediatric, 135 adult patients	Severe/very severe VOD/SOS in 173 patients, moderate VOD/SOS in 41	NR	Day +100: 62% (severe/very severe), 80% (moderate)	Acceptable safety profile
DEFIFrance [56]	251	Severe/very severe	55% (84% pediatric, 46% adult) Day +100 rate: 74% (84% in severe cases, 63% in very severe cases)	Day +100: 61% (75% in severe disease, 49% in very severe disease)	SAEs 29% Infection 17% Hemorrhage 16% Hypotension 2%
Multicenter Australian registry study [81]	111 adult, 75 pediatric: DF use in 83/73	NR	NR	Day +100: 51.8% (adult), 90.4% (pediatric)	NR
Italian AIEOP retrospective analysis [64]	103 pediatric, 67% received DF	Very severe or severe in all patients	NR	1-year survival: 61%	NR
Multi-institutional study [71]	88	100% severe, 97% MOF	36%	Day +100 35%	No worsening of clinical bleeding No grade 3/4 AEs attributed to DF
Expanded access study [65]	82 non-transplant-associated VOD/SOS	38 VOD/SOS with MOD, 44 without MOD	NR	Day +70: 74.1% (65.8% with MOD, 81.3% without MOD)	66% with AEs 25% DF-related AEs 22% hemorrhagic AEs 7.3% discontinued due to DF-related AEs
Korean analysis (ASH 2024 abstract) [79]	73	40 severe, 33 very severe	39.7% (52.5% severe, 24.2% very severe)	Day +100: 34.2% (40.3% severe, 26.4% very severe)	NR
Single-center experience [60]	51 (36 adult, 15 pediatric)	Severe VOD/SOS	Day +100: 35.3%	Day +100: 56.9%	NR
Institutional series [63]	47 RR ALL receiving inotuzumab ozogamicin pre-HCT	12 VOD/SOS: 50% very severe, 25% severe, 25% mild	67%	Day +100 mortality rate: 33% vs. 14% in patients without VOD/SOS	NR
Retrospective multicenter study [62]	45	49% severe 51% mild or moderately severe	76% (50% in severe disease)	Day +100: 64% (36% in severe disease)	Coagulation abnormalities 35%
Exploratory CIBMTR analysis [75]	41 (DF) vs. 55 (no DF)	Severe	Day +100: 51% vs. 29%	Day +100: 39% vs. 31%	Day +100 acute GvHD 23% vs. 38%
DFCI/BWH experience [57]	28 post-allo HCT	11 mild-moderate-severe, 17 very severe	75%	Day +100: 64%	Hematuria 43% Epistaxis 18% Hypotension 11% Lower GI, grade III/IV pulmonary, and grade III/IV upper GI hemorrhage each 4%
UK experience [68]	27–19/8 classic/late VOD/SOS	25 very severe, 1 severe, 1 mild	NR	Day +100: 59% (58%/63% classical/late)	NR
Retrospective series [80]	23	NR	61%	Day +100: 70%	NR
Single-center analysis [76]	14	6 severe, 4 moderate, 4 mild	79% (50% severe, 100% moderate/mild)	Day +100: 79%	No significant drug-related side-effects
Retrospective study of low-dose DF [58]	9/511 patients	NR—no ventilator support or dialysis required	100%	Time to resolution: 6–20 days from onset	NR

AE, adverse event; AIEOP, Associazione Italiana di Ematologia e Oncologia Pediatrica; ALL, acute lymphocytic leukemia; allo, allogeneic; BWH, Brigham and Women’s Hospital; CIBMTR, Center for International Blood and Marrow Transplant Research; CR, complete resolution; CUP, compassionate use program; DF, defibrotide; DFCI, Dana-Farber Cancer Institute; EBMT, European Group for Blood and Marrow Transplantation; GETH/GETMON, Grupo Español De Trasplante Hematopoyetico/Grupo Español De Trasplante De Medula Osea en Niños; GI, gastrointestinal; GO, gemtuzumab ozogamicin; GvHD, graft-versus-host disease; HCT, hematopoietic cell transplant; MOD, multi-organ dysfunction; MOF, multi-organ failure; NR, not reported; RR, relapsed/refractory; SAE, serious adverse event; SOS, sinusoidal obstruction syndrome; TRAE, treatment-related adverse event; VOD, veno-occlusive disease.

**Table 2:** Clinical trials and real-world studies of DF prophylaxis for hepatic VOD/SOS.

Study	Patients	VOD/SOS Rate	Time-to-Event Analyses	Safety
Clinical Trials				
Phase 3 HARMONY trial [95]	372 (174 aged >16 years, 198 aged ≤16 years), 190 DF vs. 182 BSC	Day +30: 4% vs. 4% Any time: 14% vs. 18%	Day +30 VOD/SOS-free survival: 67% vs. 73% Day +100: 50% vs. 57%	Grade 3/4 stomatitis 29%/1% vs. 32%/1% Grade 3/4 febrile neutropenia 28%/0% vs. 30%/2% SAEs 41% vs. 35%
Phase 3 pediatric trial [93]	356, after MAC and auto/allo HCT	Day +30: 12.2% vs. 19.9% Infants 19.6% vs. 26.8% Children 11.0% vs. 16.8% Adolescents 7.0% vs. 20.0%	Median time from HCT to VOD: 17.5 vs. 14.0 days	22% vs. 21% cumulative hemorrhage incidence 3% vs. 6% fatal infections/infestations 3% vs. 4% TA-TMA through day 180 post-HCT
Meta-analysis [94]	3005 patients, 20 studies	5% (DF total) vs. 16% (controls, 8 studies)	NR	Safety results generally consistent with known DF safety profile
Pediatric study in beta thalassemia [96]	57	1.8%	NR	DF well tolerated
Real-world studies				
Turkish retrospective analysis [105]	1153 patients	8% vs. 66.7% in high-risk patients with vs. without DF	NR	NR
DEFIFrance [56]	381 (178 pediatric, 203 adult)	20% (28% pediatric, 13% adult) by day +30	NR	SAEs 25% Hemorrhage 14% Infection 13%
Single-center experience [100]	334 high-risk pediatric allo-HCT	5.1% (*n* = 17; 4 moderate, 13 mild cases)	NR	NR
Spanish GETH/GETMON analysis [66]	253 pediatric, 135 adult patients; DF as prophylaxis in 135	NR	Day +100 survival: 89%	Acceptable safety profile
Single-center retrospective analysis [97]	237 (DF) vs. 241 (non-DF) patients undergoing HCT	0% vs. 4.8%	1-year EFS: 38% vs. 28%	Acute GvHD: 31% vs. 42%
Korean retrospective analysis [104]	69 DF vs. 78 historical controls	4.3% vs. 12.8% (2.9% vs. 28.6% in second HCT group)	0 vs. 3 VOD/SOS-related mortality	NR
Single-center series [103]	63 high-risk adult patients	6.3% (2 cases within 21 days post-HCT, 2 late-onset cases)	2-year OS 56.5% 2-year non-relapse mortality 22.3%	Bleeding 21.5% DF discontinuation 6.3% Grade II–IV acute GvHD 22.2% TA-TMA 3.2%
Single-center analysis [98]	58	0%	Day +100 survival: 100%	No hemorrhagic complications secondary to DF
Single-center experience [101]	56 adult allo-HCT	Day +30: 1.9%	1 death due to MOF at day +20 after very severe VOD/SOS	NR
Korean retrospective analysis [102]	49 (34 high-risk)	2%	Day +100 transplant-related mortality: 0%	No DF-related grade 3/4 toxicity No worsening of clinical bleeding

Allo, allogeneic; BSC, best supportive care; DF, defibrotide; EFS, event-free survival; GETH/GETMON, Grupo Español De Trasplante Hematopoyetico/Grupo Español De Trasplante De Medula Osea en Niños; GvHD, graft-versus-host disease; HCT, hematopoietic cell transplant; MAC, myeloablative conditioning; MOF, multi-organ failure; NR, not reported; OS, overall survival; SAE, serious adverse event; SOS, sinusoidal obstruction syndrome; TA-TMA, transplant-associated thrombotic microangiopathy; VOD, veno-occlusive disease.

## Data Availability

Not applicable.
